# Diagnosing coronary artery disease by sound analysis from coronary stenosis induced turbulent blood flow: diagnostic performance in patients with stable angina pectoris

**DOI:** 10.1007/s10554-015-0753-4

**Published:** 2015-09-03

**Authors:** Simon Winther, Samuel Emil Schmidt, Niels Ramsing Holm, Egon Toft, Johannes Jan Struijk, Hans Erik Bøtker, Morten Bøttcher

**Affiliations:** Department of Cardiology, Institute of Clinical Medicine, Aarhus University Hospital, Palle Juul-Jensens Boulevard 99, 8200 Aarhus, Denmark; Department of Health Science and Technology, Aalborg University, Aalborg, Denmark; College of Medicine, Qatar University, Doha, Qatar; Department of Internal Medicine, Hospital Unit West, Herning, Denmark

**Keywords:** Coronary artery disease, Angina pectoris, Heart sounds, Cardiovascular diagnostic technic, Sensitivity and specificity

## Abstract

Optimizing risk assessment may reduce use of advanced diagnostic testing in patients with symptoms suggestive of stable coronary artery disease (CAD). Detection of diastolic murmurs from post-stenotic coronary turbulence with an acoustic sensor placed on the chest wall can serve as an easy, safe, and low-cost supplement to assist in the diagnosis of CAD. The aim of this study was to evaluate the diagnostic accuracy of an acoustic test (CAD-score) to detect CAD and compare it to clinical risk stratification and coronary artery calcium score (CACS). We prospectively enrolled patients with symptoms of CAD referred to either coronary computed tomography or invasive coronary angiography (ICA). All patients were tested with the CAD-score system. Obstructive CAD was defined as more than 50 % diameter stenosis diagnosed by quantitative analysis of the ICA. In total, 255 patients were included and obstructive CAD was diagnosed in 63 patients (28 %). Diagnostic accuracy evaluated by receiver operating characteristic curves was 72 % for the CAD-score, which was similar to the Diamond–Forrester clinical risk stratification score, 79 % (*p* = 0.12), but lower than CACS, 86 % (*p* < 0.01). Combining the CAD-score and Diamond–Forrester score, AUC increased to 82 %, which was significantly higher than the standalone CAD-score (*p* < 0.01) and Diamond–Forrester score (*p* < 0.05). Addition of the CAD-score to the Diamond–Forrester score increased correct reclassification, categorical net-reclassification index = 0.31 (*p* < 0.01). This study demonstrates the potential use of an acoustic system to identify CAD. The combination of clinical risk scores and an acoustic test seems to optimize patient selection for diagnostic investigation.

## Introduction

In patients presenting with symptoms suggestive of stable angina pectoris, several diagnostic strategies can be used to obtain a correct diagnosis. Primary clinical risk stratification, e.g. the updated Diamond–Forrester score, is often performed for patient selection to non-invasive imaging or invasive coronary angiography [1]. However, the growing concern regarding increasing health expenses has increased the need for cost-effective diagnostic strategies for diagnosing coronary artery disease (CAD).

The detection of diastolic murmurs from post-stenosis coronary turbulence reported already in the 1960s was proposed for safe, cost-effective, and easy non-invasive evaluation of patients with suspected CAD [2]. Advances in computer and acoustic technology have facilitated the automated detection and analysis of diastolic heart sounds from which a risk assessment of CAD is calculated. Several research groups are currently involved in establishing signal processing techniques and coronary artery microbruit interpretation tools that are expected to increase the diagnostic accuracy of these new acoustic sensors.

Correct classification of a patients risk for CAD with an acoustic sensor or combination of clinical risk stratification scores and acoustic sensor results may not only reduce health expenses but also reduce the risk of complications related to non-invasive imaging techniques and invasive procedures.

Recently, the diagnostic accuracy of an acoustic sensor (Cardiac Sonospectrographic Analyzer model 3, SonoMedica, Virginia, United States) was reported to have a sensitivity of 90 % and specificity of 58 % compared to coronary computed tomographic angiography (CCTA) as reference standard [3]. The area under the receiver operating characteristic curves (AUC) was 74 %, which is similar or higher compared to clinical risk scorings systems [4]. In a previous study, an early algorithm and a prototype of an acoustic sensor were tested in a cohort of patient with high risk of CAD using invasive coronary angiography (ICA) as reference standard, and a diagnostic accuracy was reported with a sensitivity of 71 %, specificity of 64 %, and AUC of 77 % [5].

The primary aim of this study was to evaluate the diagnostic accuracy of an acoustic sensor with an optimized computerized algorithm and recording principle in a large cohort of patient with symptoms suggestive of stable angina pectoris. Secondarily, we compared the diagnostic accuracy of the acoustic sensor to clinical risk stratification with the updated Diamond–Forrester score, coronary artery calcium score (CACS), and their combinations.

## Materials and methods

### Study design and patients

We prospectively enrolled patients referred for CCTA or ICA as part of their evaluation of suspected obstructive CAD. Patients were recruited consecutively at a single center. Inclusion criteria were symptoms suggestive of stable angina pectoris and age above 18 years. Exclusion criteria were unstable angina pectoris or acute coronary syndrome, arrhythmia including atrial fibrillation and tachycardia higher than 85 bpm, known diastolic cardiac murmur, left ventricle ejection fraction <50 %, previous thoracic and cardiac surgery, severe chronic obstructive lung disease or asthma with inability to perform a breath hold for 8 s, active treatment for cancer or organ transplantation, and pregnancy. The study was approved by the Danish Data Protection Agency, the Central Denmark Region Committees on Health Research Ethics, and followed the principles in the declaration of Helsinki. Written informed consent was obtained from all patients.

**Fig. 1:**
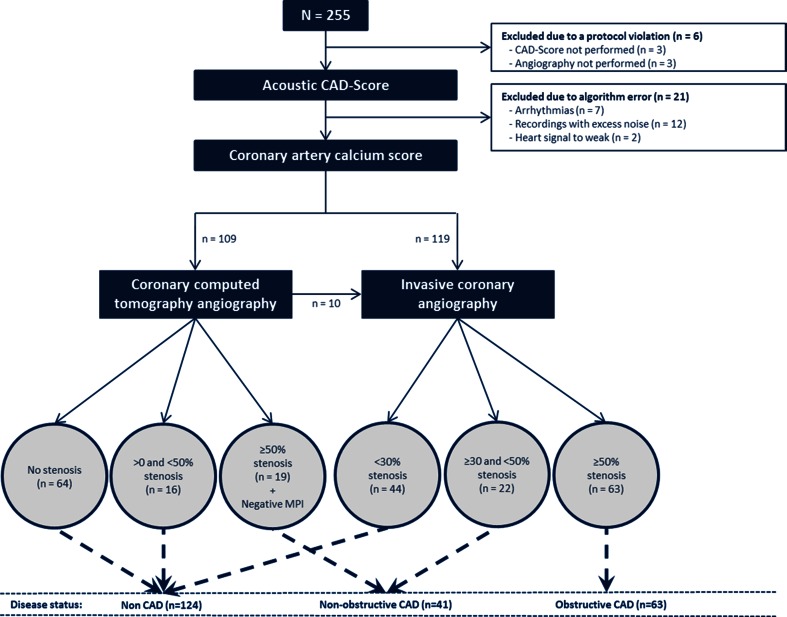
Flow chart of patients in the study. Myocardial perfusion imaging (SPECT/PET), *CAD* Coronary artery disease

All patients were scheduled for (1) a clinical visit during which an acoustic recording was obtained, (2) CACS, and (3) CCTA or ICA. In the event of an abnormal CCTA, patents were referred for myocardial perfusion imaging with single-photon emission computed tomography (99mTc-sestamibi) or positron emission tomography (^82^Rb) and subsequently to ICA if myocardial ischemia was detected (Fig. 1).

Clinical information was obtained through patient interviews and reviews of medical records. Left ventricular ejection fraction was evaluated by echocardiography.

### The CAD-sore recording and algorithm

**Fig. 2:**
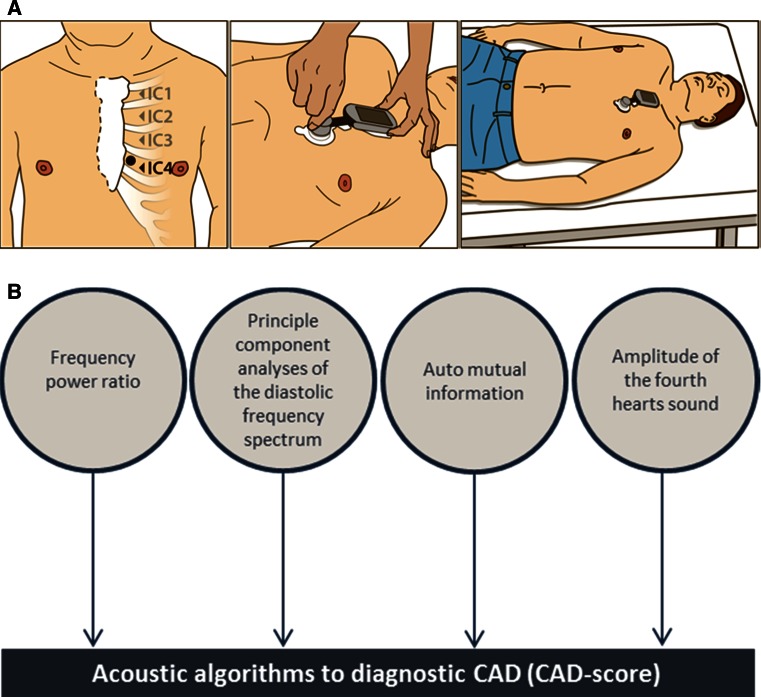
Schematic drawing of the placement and 3-min recording procedure with the CAD-score acoustic sensor system (**a**) and principle of the automatic algorithm used to calculate the CAD-score (**b**)

The acoustic sensor system recording site is the fourth left intercostal space. The recording time is 3 min, and patients were asked to hold their breath 4 times for 7.5 s (Fig. 2a). The automatic algorithm identifies acoustic properties of the diastolic heart sound statistically related to CAD. Initial algorithms were aimed at identifying only weak high frequency (>200 Hz) murmurs related to post stenotic turbulence [6]. As a supplement, low frequency changes of the diastolic heart sounds caused by CAD have also been identified recently [7, 8]. The current CAD-score algorithm combines high and low frequency measures into a combined CAD-score.

The algorithm has two main parts: (1) a pre-processing part, which identifies the mid diastolic heart sound periods (the diastasis periods between the third and fourth heart sounds) and filters out noise from the recordings and (2) the part that estimates the CAD-score. In cases in which no S4 heart sounds were identified, the analyzing window was ended 50 ms before the S1 sound. The CAD-score is based on four measures (Fig. 2b):

- Frequency power ratio (FPR), which measures the low frequency power in the mid diastolic heart sounds.
- Principle component analysis of the diastolic frequency spectrum (PCASpec). This quantifies the complete frequency spectrum from 20 to 1000 Hz into a single measure.
- Auto-mutual information (AMI), which is a complexity measure which previously has been used for successful detection of CAD [9].
- The amplitude of the fourth heart sound (S4Amp). The fourth heart sound is known as a weak predictor of ischemic heart disease [10].

The four measures are combined into the CAD-score using a standard linear discriminant score. The FPR and PCASpec measures both require a signal interval at 128 ms; however, in some patients the diastasis interval, the period between the third and fourth heart sounds, was shorter than 128 ms. These cases were labeled patients with short diastasis, and the CAD-score was estimated using only the AMI and S4 measures. The mean frequency spectrums were only calculated in patients with a period between the third and fourth heart sounds of more than 128 ms. The CAD-score ranges from 0 to 100 CAD-score points.

### Coronary computed tomography

Computed tomography scans were acquired using a dual-source multidetector scanner (SOMATOM Definition Flash, Siemens, Germany). All included patients underwent a non-enhanced scan from which CACS were calculated with the Agatston method [11].

Patients referred for CCTA subsequently underwent a contrast-enhanced scan with prospective electrocardiogram gating and dose modulation in the systolic or diastolic phases depending on heart rate. Tube settings were dependent on patient weight, and current modulation was applied. Coronary images were reconstructed using raw data iterative reconstruction. Oral and intravenous metoprolol was administrated to obtain a heart rate of <65 bpm to optimizing CCTA images. The contrast media, Ioversol (350 mg/ml), was utilized, and all patients received glycerylnitrat (0.8 mg) sublingually just prior to the CTTA.

All coronary segments were analyzed according to standard clinical practice with the use of commercially available software (Syngo.via, Siemens, Germany). The CCTA readers were permitted to use all the available post-processing image reconstruction algorithms, including axial images, multiplanar and curved reformation, maximal intensity projection, volume-rendered techniques, and cross-sectional area analysis. A semi-quantitative scale was used to grade the extent of luminal diameter stenosis. The stenosis severity was obtained in the following manner: no stenosis: 0 % diameter reduction; mild to moderate stenosis: 1–49 % diameter reduction; and severe stenosis: 50–100 % diameter reduction. Abnormal CCTA results were defined as a segment with a diameter greater than 2 mm and a more than 50 % reduction in luminal diameter. CCTA with non-evaluable segments with a diameter greater than 2 mm were also defined as abnormal. All patients with an abnormal CCTA result were referred to a myocardial perfusion imaging test or ICA, and obstructive CAD was diagnosed on the basis of these tests. No patients or segments were excluded from the analysis.

### Invasive coronary angiography

ICA was performed using standard techniques in a clinical setting. The contrast media utilized was iodixanol (350 mg/ml). Intracoronary nitroglycerin injection (200 μg) was given prior to contrast injection. Coronary segments with a reference diameter larger than 2 mm and more than 30 % diameter stenosis were categorized as having CAD (non-obstructive or obstructive). The segments with disease were visualized in multiple planes to avoid overlapping of vessels, to minimize foreshortening, and to obtain a perpendicular view of the stenosis for further analysis. Quantitative coronary angiography (QCA) was performed on all segments with disease. Image frames were selected in the end-diastolic phase, and manual edge correction was performed when needed. Obstructive CAD was defined as more than 50 % diameter stenosis by QCA. Dedicated QCA software (QAngioXA 7.3, Medis, the Netherlands) was used for the analysis and observers were blinded to risk score, CCTA results, and CAD-score.

### Statistical analysis

Gaussian distributed variables are expressed as mean (±standard deviation (SD) or total range). Variables not Gaussian distributed are presented as median (range). Categorical variables are reported as frequencies (percentages). The unpaired Student’s *t* test and ANOVA test were used for comparisons between Gaussian distributed variables. Wilcoxon rank-sum (Mann–Whitney) test and the Chi square (χ^2^) test were used for comparisons between non-Gaussian distributed and categorical variables, respectively. Pearson and Spearman tests were used to analyze correlations of variables of Gaussian and non-Gaussian distributions, respectively. The area under the receiver operating characteristic (AUC) curve was calculated for continues variables, and the optimal cut point was established by the method described by Liu X [12]. Sensitivity, specificity, positive and negative predictive values (PPV and NPV), and positive and negative likelihood ratios (PLR and NLR) were calculated for binary variables, with quantitative ICA as reference. CAD-scores were divided as a binary variable and into three levels: low (<20), intermediate (20–30), and high (>30). Continuous net-reclassification index (NRI) and integrated discrimination improvement (IDI) were tested for evaluating the benefit of combining the Diamond-Forrester score and CAD-score. Calculation of the categorical net-reclassification index (NRI) was performed according to four predefined risk categories: very low: <10 %, low: ≥10 to <30 %, moderate: ≥30 to <60 %, and high: ≥60 %. All *p*-values are two-sided with a 5 % level of significance. Statistical analyses were performed using STATA, version 13.1 (StatCorp LP, United States), but NRI and IDI were calculated using the R package PredictABEL [13]. To validate the algorithm and avoid overfit to randomness in the current heart sound recording, the linear discriminant function, which combines the four features, was tested using 20 times tenfold cross validation [14].

## Results

We enrolled 255 patients in this study. Of these, 6 were patients excluded due to the lack of a CAD-score or angiography. Twenty-one patients were excluded due to errors in the computerized algorithm registered as arrhythmias (*n* = 7), recordings with excess noise (*n* = 12) or the heart signal was too weak (*n* = 2). Of the remaining 228 patients, 109 (48 %) patients were referred to CCTA and 119 (52 %) to ICA (Fig. 1).

Based on the results of the CCTA and ICA, the patients were grouped into non-CAD (*n* = 124), non-obstructive CAD (*n* = 41), and obstructive CAD (*n* = 63) as demonstrated in Fig. 1. Baseline and cardiac imaging characteristic are listed in Table 1.

**Table 1:** Table of baseline and the cardiac imaging characteristics in patients with non-CAD, non-obstructive CAD, and obstructive CAD

	Non CAD	Non-obstructive CAD	Obstructive CAD
Patients	124	41	63
Patients characteristic			
Age	58.9 ± 11.1	64.5 ± 9.4**	65.3 ± 9.2***
Gender (Male)	51 (41 %)	22 (54 %)	48 (76 %)***
BMI	27.4 ± 4.5	25.2 ± 2.8**	26.6 ± 4.0
Blood pressure			
Systolic	137 ± 19	145 ± 20*	143 ± 18*
Diastolic	81 ± 10	82 ± 12	82 ± 11
Heart frequent	65 ± 9	67 ± 12	65 ± 10
Smoking			*
Actively	28 (23 %)	8 (20 %)	11 (17 %)
Previous	41 (33 %)	13 (32 %)	37 (59 %)
None	54 (44 %)	19 (46 %)	15 (24 %)
Cholesterol			
Total	5.1 ± 1.1	5.1 ± 1.2	5.0 ± 1.1
LDL	3.1 ± 1.0	3.0 ± 1.0	3.1 ± 1.1
HDL	1.5 ± 0.5	1.5 ± 0.4	1.4 ± 0.4
Triglycerides	1.4 ± 0.8	1.4 ± 0.8	1.7 ± 1.0
Diabetes	8 (6 %)	4 (10 %)	9 (14 %)
Previous percutaneous coronary intervention	1 (1 %)	5 (12 %)***	17 (27 %)***
Type of symptoms			***
Non-cardiac chest pain	20 (16 %)	8 (20 %)	8 (13 %)
Atypical	70 (56 %)	19(46 %)	12 (19 %)
Typical	34 (27 %)	14 (34 %)	43 (68 %)
Diamond–Forrester score, mean	25 ± 17	34 ± 21**	51 ± 22***
Diamond–Forrester risk categories		**	***
Very low, <10 %	27 (22 %)	1 (2 %)	1 (2 %)
Low, ≥10 to <30 %	56 (45 %)	20 (49 %)	14 (22 %)
Moderate, ≥30 to <60 %	34 (27 %)	13 (32 %)	21 (33 %)
High, ≥60 %	7 (6 %)	7 (17 %)	27 (43 %)
Cardiac imaging characteristics			
Left ventricle ejection fraction by echo	61 ± 4	60 ± 4	60 ± 3
Coronary artery calcium score^‡^, mean	64 ± 147	414 ± 465***	1130 ± 1293***
Coronary artery calcium score groups		***	***
=0	70 (57 %)	2 (5 %)	2 (3 %)
>0 and <400	47 (38 %)	22 (54 %)	23 (38 %)
≥400	6 (5 %)	17 (42 %)	36 (59 %)
Coronary vessel disease by ICA			
1-Vessel disease	NA	NA	44 (70 %)
2-Vessel disease	NA	NA	14 (22 %)
3-Vessel disease or left main	NA	NA	5 (8 %)
Diseased vessel diameter by ICA^#,‡‡^			
Diameter < 3 mm	NA	NA	30 (%)
Diameter ≥ 3 mm	NA	NA	32 (%)
Stenosis diameter reduction by ICA^##^			
Stenosis ≥50 and <70 %	NA	NA	35 (56 %)
Stenosis ≥70 and <100 %	NA	NA	21 (33 %)
Stenosis = 100 %	NA	NA	7 (11 %)
Stenosis by vessel^†^			
Stenosis in LM or LAD	NA	NA	33
Stenosis in CX	NA	NA	23
Stenosis in RCA	NA	NA	28

Data are missing in 3 patients^‡^ and 1 patient^‡‡^. In the event of multivessel disease, the vessel with the largest diameter^#^ and most severe vessel diameter narrowing^##^ was registered. Patients with multivessel disease are presented more than once^†^

Statistical significance compared to the non-CAD group is showed in the table with: * if *p* < 0.05; ** if *p* < 0.01; *** if *p* < 0.001

**Fig. 3:**
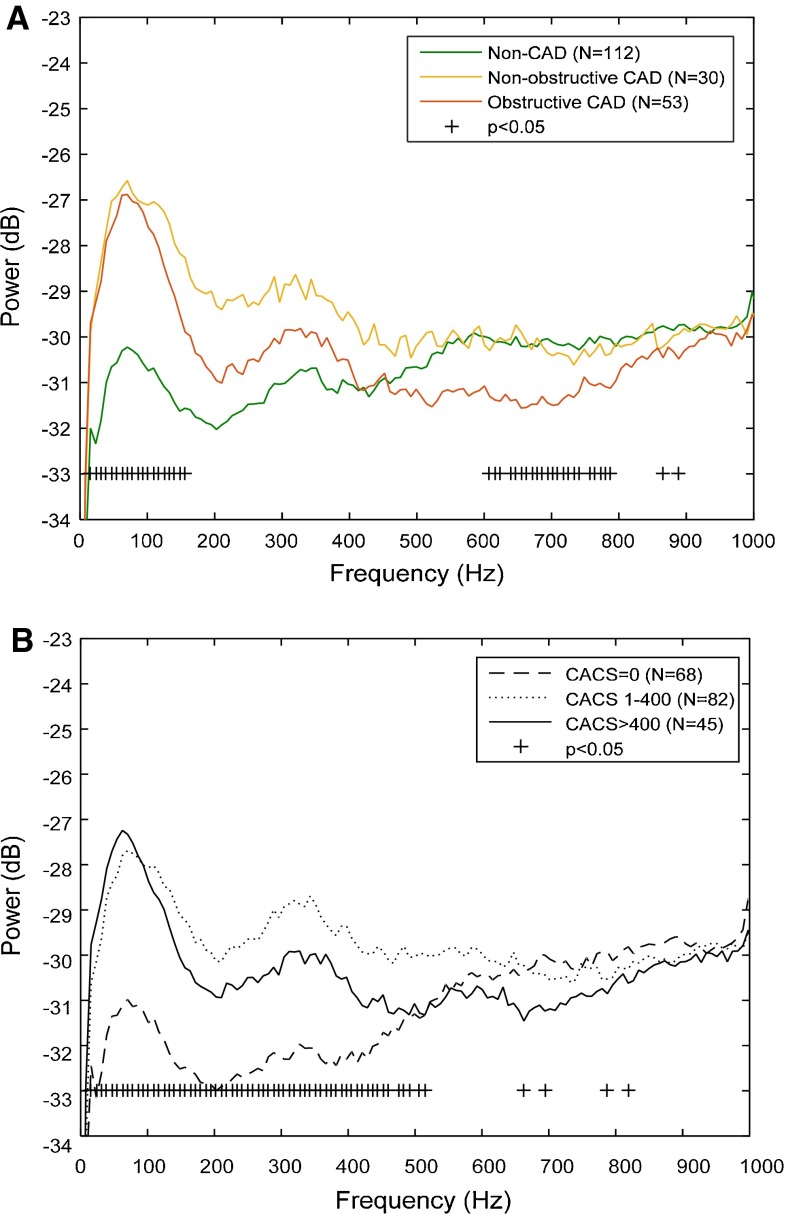
Average diastolic frequency spectrum plots which show the distribution of power across frequencies. Illustrated is the average frequencies spectrums relation to different degrees of CAD (**a**) and CACS scores (**b**). Included in the analysis are only patients with a period between the third and fourth heart sounds of more than 128 ms

Comparing the sound power (dB) and frequency (Hz), we saw a significant difference between the mean frequency spectrums in patients with non-CAD compared to patients with non-obstructive and obstructive CAD (Fig. 3a). The mean CAD-score calculated from the frequency spectrums was 21.3 ± 12.7 in the non-CAD group, which was significantly low than the CAD-score in the non-obstructive CAD group, 29.7 ± 11.8 (*p* < 0.001) and in the obstructive CAD group, 32.8 ± 10.8 (*p* < 0.001).

**Fig. 4:**
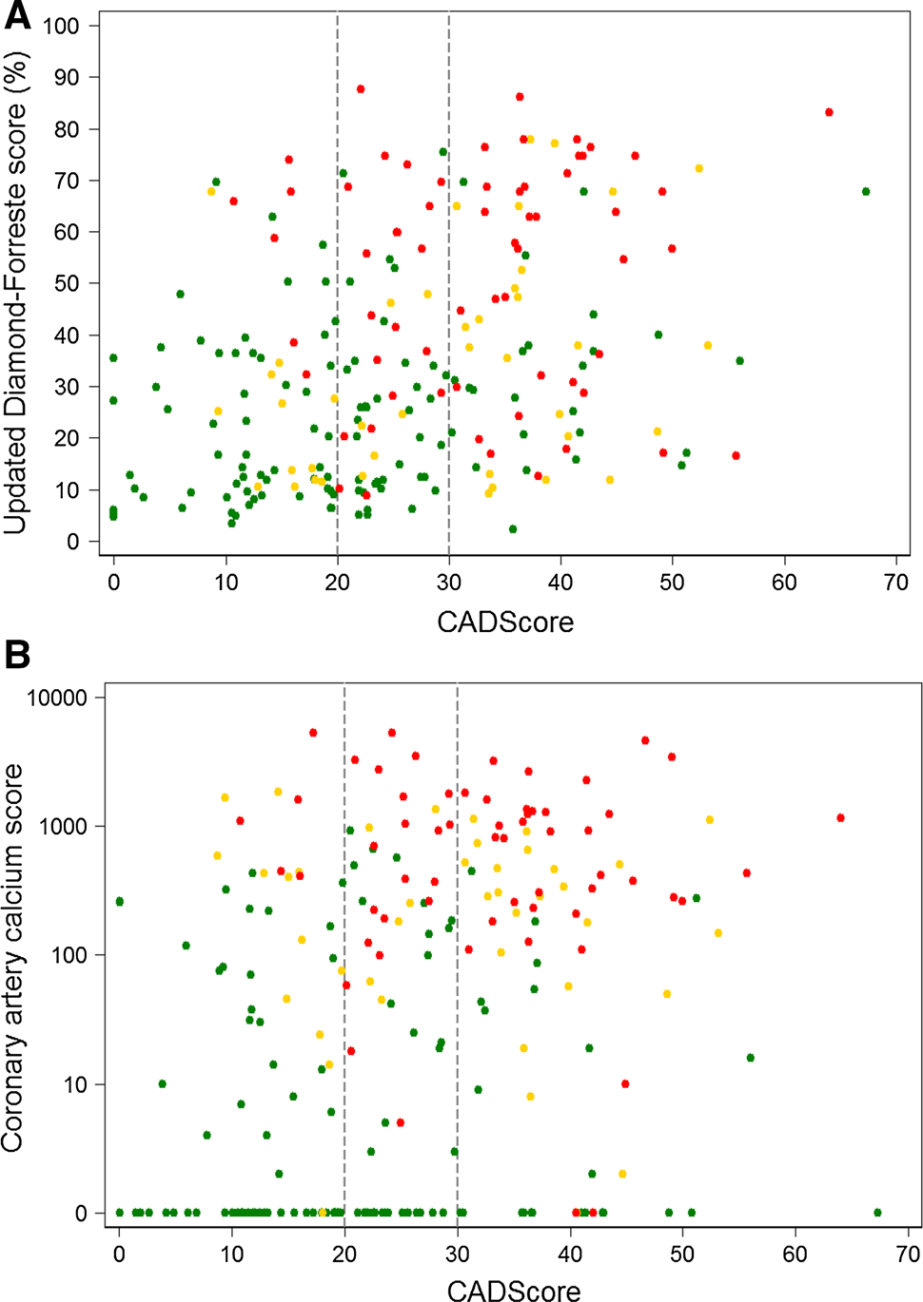
Correlation between CAD-score and the updated Diamond-Forrester score (**a**) and CAD-score and coronary artery calcium scorende (**b**). *Red dots* mark patients with obstructive coronary artery disease (*n* = 63), *yellow dots* patients with non-obstructive coronary artery disease (*n* = 41), and *green dots* patients with non-coronary artery disease (*n* = 124). The *dashed line* illustrates a CAD-score cutoff at 20 and 30

There was a weak correlation between the CAD-score and the Diamond–Forrester score, Pearson coefficient = 0.36 (*p* < 0.001) (Fig. 4a). However, CAD-scores increased in the non-obstructive CAD and obstructive CAD groups compared to the non-CAD group even when patients were divided into clinical risk stratification categories and CACS groups (Table 2).

**Table 2:** Table of mean CAD-scores according to CAD status

	Non CAD	Non-obstructive CAD	Obstructive CAD
CAD-score, mean	21.3 ± 12.7	29.7 ± 11.8***	32.8 ± 10.8***
Diamond-Forrester risk categories			
Very low, <10 %	15.0 ± 9.2	33.6 ± NA	22.6 ± NA
Low, ≥10 to <30 %	21.9 ± 11.7	25.8 ± 11.6	34.1 ± 10.5***
Moderate, ≥30 to <60 %	23.4 ± 13.4	32.0 ± 10.3*	30.4 ± 9.9*
High, ≥60 %	30.6 ± 19.6	34.3 ± 11.6	34.3 ± 11.6*
Coronary artery calcium score groups^‡^			
=0	20.9 ± 13.2	29.3 ± 16.0	41.3 ± 1.1*
>0 and <400	21.7 ± 12.7	31.6 ± 10.9**	33.1 ± 9.6***
≥400	21.9 ± 6.3	27.1 ± 12.9	32.5 ± 11.6*
*Invasive coronary angiography*			
Coronary vessel disease by ICA			
1-vessel disease	NA	NA	32.6 ± 10.4
2-vessel disease	NA	NA	32.8 ± 11.7
3-vessel disease or left main	NA	NA	33.9 ± 13.4
Diseased vessel diameter by ICA^#,‡‡^			
Diameter < 3 mm	NA	NA	31.5 ± 9.5
Diameter ≥ 3 mm	NA	NA	33.9 ± 12.0
Stenosis diameter reduction by ICA^##^			
Stenosis ≥50 and <70 %	NA	NA	33.2 ± 10.5
Stenosis ≥70 and <100 %	NA	NA	33.1 ± 12.0
Stenosis = 100 %	NA	NA	29.5 ± 8.3
Stenosis by vessel^†^			
Stenosis in LM or LAD	NA	NA	31.4 ± 11.9
Stenosis in CX	NA	NA	35.4 ± 11.5
Stenosis in RCA	NA	NA	31.9 ± 8.3

Data are missing in 3 patients^‡^ and 1 patient^‡‡^. In the event of multivessel disease, the vessel with the largest diameter^#^ and most severe vessel diameter narrowing^##^ was registered. Patients with multivessel disease are presented more than once^†^

Statistical significance compared to the non-CAD group is showed in the table with: * if *p* < 0.05; ** if *p* < 0.01; *** if *p* < 0.001

Similarly, the correlation between CAD-score and CACS was weak, Spearman´s rho = 0.28 (*p* < 0.001) and the CAD-score increased significantly between the groups in patients with CACS at 0, higher than 0 but lower than 400, and higher than 400 (Fig. 4b; Table 2). A significant mean difference between the mean frequency spectrums was also seen in patients with CACS 0, higher than 0 but lower than 400, and higher than 400 (Fig. 3b).

There was no significant difference in CAD-score between patients with single vessel obstructive CAD compared to patients with multivessel obstructive CAD. In addition, no differences in CAD-score were found between patients with vessels with a reference lumen diameter larger than 3 mm compared to smaller than 3 mm or diameter stenosis narrowing between 50 and 70 %, 70 and 99 %, or 100 % (Table 2). Five of the seven patients with a 100 % occluded stenosis did not have other significant stenosis. In these, the average CAD-score was 28.5, which is lower than the average in the obstructive CAD group, but higher than in the non-CAD group.

### Diagnostic accuracy

**Fig. 5:**
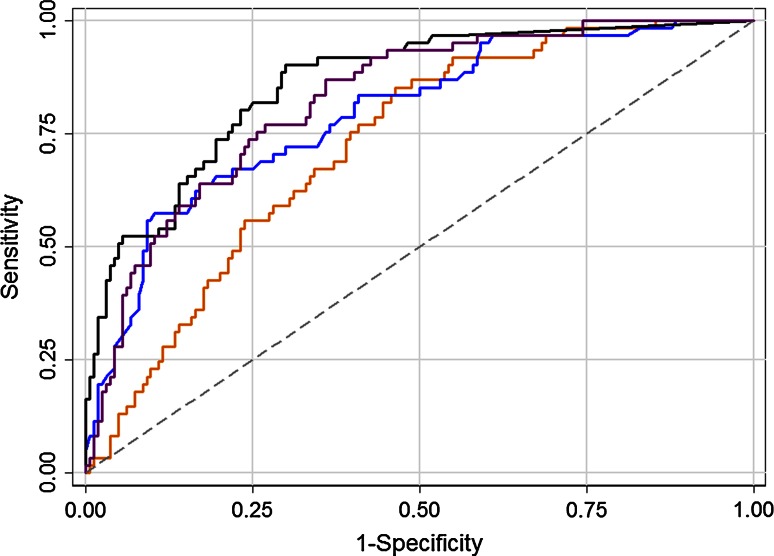
Receiver operating characteristic curve for CAD-score [*orange line*, AUC: 72 % (CI 65–79)], Diamond-Forrester score [*blue line*, AUC: 79 % (CI 72–86 %)], coronary artery calcium score [*black line*, AUC: 86 % (CI 81–91 %)] and the combined Diamond-Forrester score and CAD-score [*purple line*, AUC 82 % (CI 76–88 %)]. The *grey dash line* is the reference line

The diagnostic accuracy of obstructive CAD vs. non-obstructive and non-CAD evaluated by AUC was for the CAD-score, 72 % (CI 65–79 %), which was non-significantly lower compared to the Diamond-Forrester Score, 79 % (CI 72–86 %) (*p* = 0.12) and significantly lower than CACS, 86 % (CI 81–91 %) (*p* < 0.01). When the CAD-score and Diamond-Forrester score were combined, AUC increased to 82 % (CI 76–88 %), which was significantly higher compared to both standalone CAD-score (*p* < 0.01) and the Diamond-Forrester score (*p* < 0.05). The combination of CAD-score and the Diamond-Forrester Score was not significantly lower than CACS alone (*p* = 0.28) (Fig. 5). There was a limited benefit from combining CAD-score and the Diamond-Forrester score with CACS or combining all three scores together, AUC: 87 % (CI 82–92 %), 87 % (CI 82–92 %) and 89 % (CI 84–93 %), respectively. When the algorithm was adjusted using the tenfold cross-validation scheme, the AUC was 70.5 %, which was close to the 72 % obtained by the final CAD-score, indicating a low risk of overfitting.

The optimal CAD-score threshold for a binary prediction of obstructive CAD was 24.2. Using this threshold, the sensitivity, specificity, PPV, and NPV were 76 % (CI 64–86 %), 59 % (CI 52–67 %), 42 % (CI 33–51 %) and 87 % (CI 79–92 %), respectively.

A low CAD-score (≤20) was observed in 62 (50 %) non-CAD patients, 12 (29 %) non-obstructive CAD patients, and 6 (10 %) CAD patients. An intermediate score was observed in 37 (30 %) non-CAD patients, 6 (15 %) non-obstructive CAD patients and in 20 (32 %) CAD patients. The remaining 25 (20 %) of the non-CAD patients, 23 (56 %) of the non-obstructive CAD patients, and 37 (59 %) CAD patients had a high CAD-score (>30). NPV of a low CAD-score (≤20) was 92.5 % (CI 87–98 %), and the PPV of a high CAD-score (>30) was 43.5 % (CI 33–54 %) when both non-CAD and non-obstructive CAD patients were considered healthy.

Continuous-NRI calculated for the diagnostic strategy of adding CAD-score to the Diamond-Forrester score was 0.71 (CI 0.50–0.92) (*p* < 0.001) and IDI was 0.09 (CI 0.04–0.14) (*p* < 0.001). Categorical-NRI calculated for the diagnostic strategy of adding the CAD-score to the Diamond-Forrester score reclassified 18 patient with obstructive CAD to a higher risk category and 6 patients to a lower risk category. Of patients without obstructive CAD, 55 patients were reclassified to a lower risk category and 36 to a higher category. Categorical NRI was 0.31 (CI 0.12–0.49) (*p* < 0.01) (Table 3).

**Table 3:** Table of the risk stratification with updated Diamond–Forrester score (DF-score) and with the combined model of Diamond–Forrester score and CAD-score

	Prevalence of CAD in a model combining DF-score and CAD-score				Total
	<10 %	≥10 to <30 %	≥30 to <60 %	≥60 %	
Numbers of patients and prevalence of CAD (%)					
DF-score: <10 %	26 (4 %)	3 (0 %)	0 (0 %)	0 (0 %)	29 (3 %)
DF-score: ≥10 to <30 %	34 (0 %)	31 (19 %)	25 (32 %)	0 (0 %)	90 (16 %)
DF-score: ≥30 % to <60 %	10 (10 %)	11 (18 %)	21 (38 %)	26 (38 %)	68 (31 %)
DF-score: ≥60 %	0 (0 %)	4 (50 %)	2 (50 %)	35 (69 %)	41 (66 %)
Total	70 (3 %)	49 (20 %)	48 (35 %)	61 (56 %)	228
Obstructive CAD					
DF-score: <10 %	1	0	0	0	1
DF-score:≥10 to <30 %	0	6	8	0	14
DF-score: ≥30 to <60 %	1	2	8	10	21
DF-score: ≥60 %	0	2	1	24	27
Total	2	10	17	34	63
Non or non-obstructive CAD					
DF-score: <10 %	25	3	0	0	28
DF-score: ≥10 to <30 %	34	25	17	0	76
DF-score: ≥30 to <60 %	9	9	13	16	47
DF-score: ≥60 %	0	2	1	11	14
Total	68	39	31	27	165
*Net-reclassification index—categorical*					
Obstructive CAD patients					
Classified upward: 18 (29 %)					
Classified downward: 6 (10 %)					
Classified into a more relevant risk class: 18 – 6 = 12 (19 %)					
Non or non-obstructive CAD patients:					
Classified upward: 36 (22 %)					
Classified downward: 55 (33 %)					
Classified into a more relevant risk class: 55 – 36 = 20 (12 %)					
Calculation of categorical net-reclassification index					
Patients in total, classified into a more relevant risk class: 19 % + 12 % = 31 % (*p* ≤ 0.01)					

Reclassification of patients with the combined model Diamond–Forrester score and CAD-score is showed compared to Diamond–Forrester score alone, and categorical net-reclassification index is calculated

In total, 70 (31 %) patients were classified in the very low risk category with the diagnostic model including both Diamond-Forrester score and CAD-score compared to 29 (13 %) patients with the Diamond-Forrester Score. The disease prevalence in the very low risk category was 3 % for both models. The combined diagnostic model classified 58 in the high risk category compared to 39 with the Diamond-Forrester score, and the disease prevalence decreased to 55 % from 64 %.

## Discussion

The main finding in this study was that an acoustic sensor providing the CAD-score seems to predict obstructive CAD independent of the updated Diamond-Forrester score and CACS in patients presenting with symptoms suggestive of stable angina pectoris. We demonstrated an additive diagnostic accuracy when combining the Diamond-Forrester score and the CAD-score. Of particular interest, the high number of patients reclassified to the very low risk category indicates the clinical potential of this novel diagnostic method in primary risk stratification before non-invasive and invasive coronary diagnostic procedures.

### Acoustic sensor and algorithm

The acoustic CAD-score is based on heart sound recordings obtained with an ultra-sensitive microphone and a novel signal-processing algorithm. The novelty of the algorithm is the combination of information from both the low and high frequency parts of the diastolic heart sounds. The high frequency feature quantifies high frequency microbruits, which are expected to occur at frequencies above 200 Hz [6]. However Fig. 4a demonstrates a significant drop in absolute frequencies above approximately 500 Hz, which may indicate that the diastolic energy concentrates at lower frequencies below 500 Hz. However the microbruits are of low amplitude, and the variation in absolute power across subjects is large, therefore the microbruits might very well be buried in the absolute frequency spectrums. The significant increase in power below 150 Hz, see Fig. 4a, is in line with recent findings [7, 8]. The cause of the increased diastolic heart sound pressure at lower frequencies is unknown, but the low frequency sound is likely due to oscillations in the myocardium. These oscillations might occur due to altered diastolic filling patterns or changes in resonate frequencies of the coronary artery system due to stiffening of the arteries.

The diagnostic performance of the acoustic CAD-score is in line with the performance (AUC 74.3 %) reported by Makaryus AN et al. [3] of the Cardiac Sonospectrographic Analyzer. However, contradictory to the Cardiac Sonospectrographic Analyzer, the current system is based on a single recording from a recording site at the 4th intercostal room, which may simplify the use of the system.

### Diagnostic accuracy

In the present study we compared the diagnostic accuracy of the acoustic CAD-score to a simple clinical risk stratification score based on age, gender, and type of chest symptoms. As a standalone test, the CAD-score performed as well as the updated Diamond-Forrester score. However, a significantly increased diagnostic accuracy was detected when combining the two scores. This is an improvement over the lack of increased diagnostic accuracy by adding more advance clinical risk scores, such as the Duke risk score (based on sex, age, diabetes, tobacco use, history of myocardial infarction, and symptoms of angina pectoris) or Morise risk score (based on sex, age, diabetes, tobacco use, symptoms of angina pectoris, hypercholesterolemia, hypertension, family history of CAD, obesity, and estrogen status) [4, 15, 16].

CACS has in previous studies demonstrated high diagnostic accuracy of CAD when evaluated by AUC. Interestingly, we only found a weak correlation between the CACS and the CAD-score. This finding is similar to the correlation between CACS and results obtained by the Cardiac Sonospectrographic Analyzer [3]. This may indicate that acoustic tests of CAD measures coronary stenosis severity rather than artery wall stiffness.

The simple and non-invasive method without contrast or radiation exposure described here seems to be an attractive approach to risk stratification of patients with symptoms suggestive of stable angina pectoris. Nonetheless, our study demonstrated only moderate diagnostic accuracy of this novel test as standalone modality. Interestingly, a high negative predictive value of a low CAD-score value (≤20) indicated a potential as a rule-out device. Thus, as add-on to clinical risk stratification, diagnosis of CAD by sound analysis from coronary stenosis-induced turbulent blood flow may have a clinical role, e.g. in primary care settings.

## Limitations

Since the CAD-score is based on diastolic heart sounds, subjects with diastolic murmurs were not included in the study. This means that auscultation before estimating the CAD-score is required to rule out diastolic murmurs due to valvular heart disease. Data from the current study population were used for both development and validation of the acoustic CAD-score. This implies a risk of overfitting the algorithm to the current population. To minimize this risk, the cross-validation was applied and showed similar performance results, indicating a low risk of overfitting. However, further validation in a prospective study has to be performed to confirm the current findings.

## Conclusion

This study demonstrates the potential use of a non-invasive, non-radiation acoustic detection system to identify coronary artery disease. The combination of clinical risk scores and an acoustic test seems to optimize patient selection for diagnostic investigation.
